# Case report: Pragmatic impairment in multiple sclerosis after worsening of clinical symptoms

**DOI:** 10.3389/fpsyg.2022.1028814

**Published:** 2022-11-24

**Authors:** Sara Lago, Francesca Bevilacqua, Maria Rosaria Stabile, Cristina Scarpazza, Valentina Bambini, Giorgio Arcara

**Affiliations:** ^1^IRCCS San Camillo Hospital, Venice, Italy; ^2^Department of Neuroscience, Padova Neuroscience Centre, University of Padova, Padua, Italy; ^3^Department of Neurology, ULSS2 Marca Trevigiana, Conegliano, Italy; ^4^Department of General Psychology, University of Padova, Padua, Italy; ^5^Department of Humanities and Life Sciences, University School for Advanced Studies IUSS, Pavia, Italy

**Keywords:** pragmatics, pragmatic impairment, multiple sclerosis, language, communication, case report

## Abstract

Pragmatics, defined as the ability to integrate language and context to communicate effectively, may be impaired in Multiple Sclerosis (MS). We present the case of a patient with active secondary progressive MS who, after a first neuropsychological assessment that evidenced only a slight pragmatic impairment, suffered a sudden worsening of her clinical conditions, treated with corticosteroids. After this clinical worsening, her pragmatic abilities declined markedly, both in comprehension and production. This worsening was accompanied by a decline only in one attention task, in the context of an overall stable cognitive functioning. We conclude that pragmatics may be a domain particularly susceptible to cognitive worsening, highlighting the importance of its assessment in clinical practice.

## Introduction

Multiple sclerosis (MS) is an autoimmune disease of the nervous system, characterized by damage to the myelin sheath of the white matter fibers. This damage affects neural transmissions, resulting in a range of physical and cognitive symptoms. MS is associated with cognitive impairment in 43%–72% of patients, who show deficits, especially in executive functions, processing speed, visual and verbal learning, memory, attention, and working memory (Chiaravalloti and DeLuca, 2008). Moreover, MS patients may show impairment also in language and communication abilities (Sonkaya and Bayazit, 2018; Feenaughty, 2022). More in detail, a review by Renauld et al. (2016) showed that MS is commonly associated with impaired performance in verbal fluency and sentence comprehension tasks, and later studies found that communication difficulties in MS are related to impairments in processing speed (Yap et al., 2022) and in executive functioning (Delgado-Álvarez et al., 2021). This evidence suggests an association between cognitive and linguistic domains.

While cognitive impairments in MS are largely considered in research and clinical practice, there is a tendency to overlook language difficulties, and in particular, those aspects related to communication, such as the ability to have a conversation or, in general, to convey or understand the intended meaning depending on context (Sonkaya and Bayazit, 2018). These abilities are typically considered as belonging to the domain of pragmatics, which supports the flexible and ecological use of language (Cummings, 2021). In the last decades, pragmatics has become a topic also for neuropsychological assessment, and several clinical batteries have been developed to assess impairment in the pragmatic domain (Bosco et al., 2012; Arcara and Bambini, 2016). Nevertheless, only a few studies so far have addressed the pragmatic impairment in MS. A study on patients with relapsing–remitting and progressive MS (Carotenuto et al., 2018a) showed that about 55% of the enrolled participants exhibited some deficit in pragmatic tasks, with the deficit being present both in cognitively impaired and unimpaired patients yet showing significant associations with executive functions and social cognition. Another study showed that the pragmatic impairment was strongly tied to the neural connectivity involving bilateral temporoparietal regions (Carotenuto et al., 2018b). In addition, earlier studies reported that MS patients have difficulties in understanding ambiguous sentences and metaphors (Lethlean and Murdoch, 1997) and that they have impaired language production, specifically regarding flexibility in complex discourse; these difficulties were found to be related to cognitive impairment, particularly to executive dysfunction (Arrondo et al., 2010), in line with the most recent data (Carotenuto et al., 2018a). Despite the limited number of studies, evidence points to a relevant deficit of pragmatics in MS (Carotenuto et al., 2021), and to an association between pragmatic deficits and other cognitive abilities, especially executive functions.

Here, we present the case of PM, a 63-year-old female patient (with 8 years of education) with active secondary progressive MS (SPMS; Lublin et al., 2014). When she first came to our attention, the neuropsychological assessment showed an isolated subtle pragmatic impairment, not accompanied by other cognitive deficits based on a traditional neuropsychological test battery. Later, PM suffered a serious worsening of her clinical conditions, treated with corticosteroids. Before discharge from the hospital, she underwent a second assessment, which showed a diffuse pragmatic deficit accompanied by a decline in attention.

## Materials and methods

**Figure 1:**
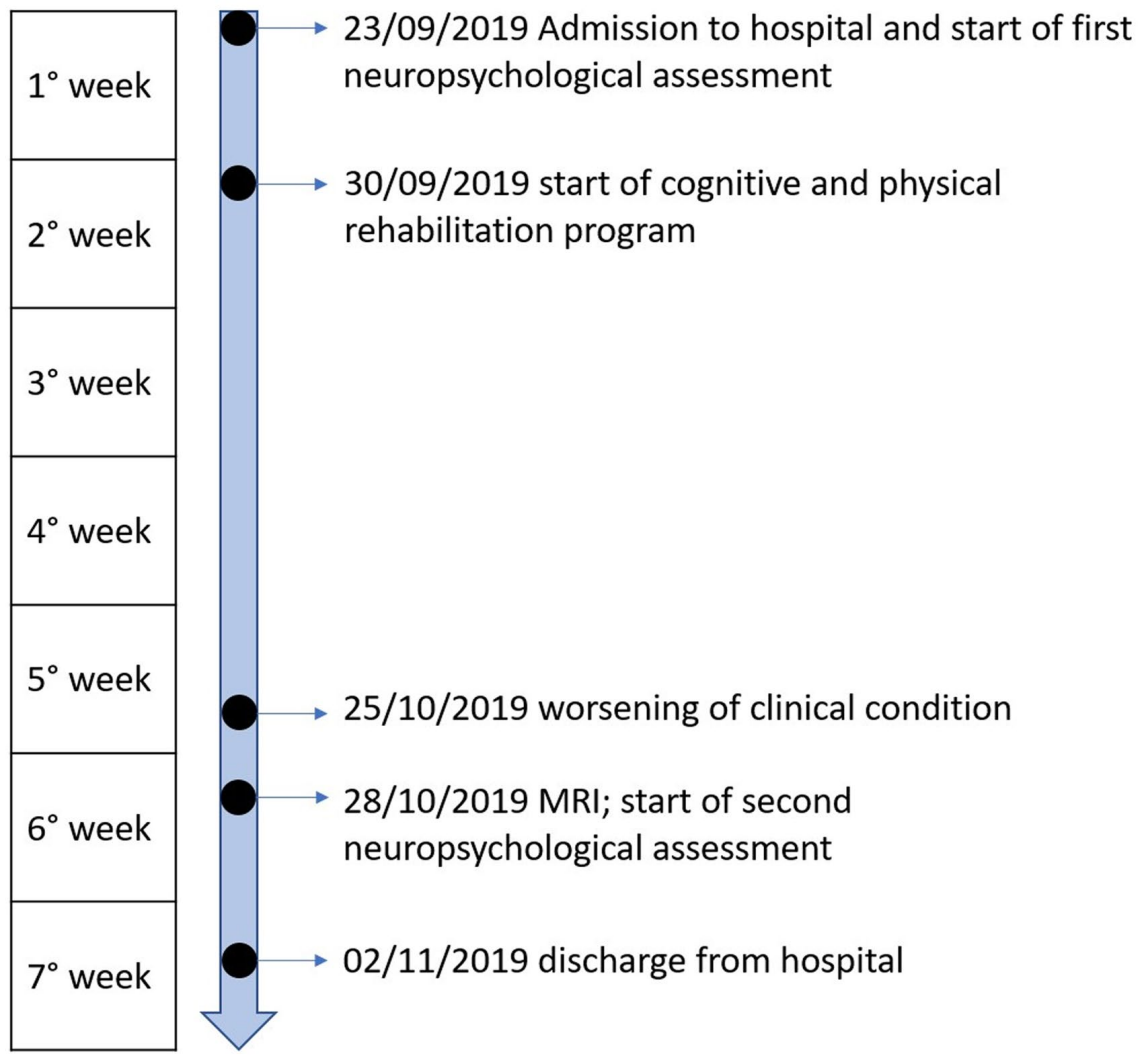
Timeline of events of the case study.

PM was first diagnosed with relapsing–remitting MS, which later evolved into SPMS, about 25 years before testing. In 2019, the disease was active and progressing, so PM was admitted to the hospital for intensive physical rehabilitation since her main complaints were mostly fatigue and walking impairment. PM gave informed consent for her data (except MR scan images) to be used in this study. Hospitalization lasted approximately 7 weeks from the admission date (see Figure 1). Upon admission, PM underwent a first thorough neuropsychological assessment, covering pragmatics and a wide range of cognitive domains (see Table 1). The patient was on teriflunomide therapy and her Expanded Disability Status Scale (EDSS; Kurtzke, 1983) score was 6.5. Neuropsychological tests, described more in detail in Supplementary material, included:

- Brief International Cognitive Assessment for Multiple Sclerosis (BICAMS; Italian version by Goretti et al., 2014);
- Assessment of Pragmatic Abilities and Cognitive Substrates (APACS; Arcara and Bambini, 2016);
- Test of Reception of Grammar 2 (Bishop, 2003);
- WAIS Vocabulary (Orsini and Pezzuti, 2013);
- Naming on Verbal Description (Novelli et al., 1986);
- Phonemic and Semantic Fluency (Novelli et al., 1986);
- Rey Auditory Verbal Learning Test (RAVLT; Carlesimo et al., 1996);
- Forward Digit Span (Monaco et al., 2013);
- Backward Digit Span (Monaco et al., 2013);
- Corsi Blocks Forward Span (Monaco et al., 2013);
- Corsi supraspan (Spinnler and Tognoni, 1987);
- Story-based Empathy Task (Dodich et al., 2015);
- Pyramids and Palm Trees Test (Gamboz et al., 2009);
- Attentional Matrices (Della Sala et al., 1992);
- Clock Drawing Test (Mondini et al., 2011);
- free hand-copying of drawings (Carlesimo et al., 1996);
- Raven’s Colored Progressive Matrices (CPM, Carlesimo et al., 1996);
- Trail Making Test—A and B (Giovagnoli et al., 1996);
- Psychological Well-Being scales (Italian version by Ruini et al., 2003);
- Communication Outcome after Stroke (Italian version by Bambini et al., 2017).

**Table 1:** Neuropsychological tests.

Domain	Test	First assessment raw (and corrected) scores	Second assessment raw (and corrected) scores	Cut-off	Maximum score	Thresholds for significant changes (APACS only)	First assessment percentage − transformed score	Second assessment percentage − transformed score	Magnitude of percentage score difference (second − first assessment)
Pragmatics	APACS interview	34^*^	31^*^	42	44	No thresholds available	77.27	70.45	−6.82
	APACS description	46	41^*^°	45	48	44—no upper threshold	95.83	85.42	−10.42
	APACS narratives	47	39^*^°	47	56	52—no upper threshold	83.93	69.64	−14.29
	APACS figurative language 1	14	14°	13	15	14—no upper threshold	93.33	93.33	0
	APACS humor	5	7°	4	7	5–6	71.43	100	+28.57
	APACS Figurative language 2	13^*^	10^*^	21		No thresholds available	43.33	33.33	−10
	APACS pragmatic comprehension composite	0.73^*^	0.74^*^	0.78	1	0.72–0.84	73	74	+1
	APACS pragmatic production composite	0.87^*^	0.78^*^°	0.95	1	0.87–0.92	87	78	−9
	APACS total composite	0.8^*^	0.76^*^°	0.88	1	0.82–0.88	80	76	−4
General MS cognitive screening	Symbol digit modalities testing (SDMT)	29 (36.7)	32 (40.5)	35	110	-	26.36	29.09	+2.73
	California verbal learning test-II (CVLT-II)	57 (58.6)	54 (55.1)	35	80	-	71.25	67.50	−3.75
	Brief visuospatial memory test-revised (BVMT-R)	7 (38.1)	12 (44.5)	35	36	-	19.44	33.33	+13.89
Language	Test of reception of grammar 2 (TROG-2)—blocks G, S, K, T	13	14	NA	16	-	81.25	87.50	+6.25
	TROG-2—vocabulary	47	47	NA	48	-	97.92	97.92	0.00
	WAIS vocabulary	33 (8)	34 (8)	NA	70	-	47.14	48.57	+1.43
Executive functions supported by language	Naming on verbal description	25 (25)^*^	29 (29)^*^	33.25	38	-	65.79	76.36	+10.53
	Phonemic fluency	15 (18)	19 (22)	16	34	-	44.12	55.88	+11.76
	Semantic fluency	30 (32)	31 (33)	25		-	88.24	91.18	+2.94
Verbal memory	Rey auditory verbal learning test (RAVLT), immediate	44 (46.3)	49 (51.3)	28.53	75	-	58.67	65.33	+6.66
	RAVLT, delayed	10 (10.07)	Not completed	4.69	15	-	66.67		
Short-term memory	Forward digit span	5 (5)	6 (6)	3.75	9	-	55.56	66.67	+11.11
	Corsi blocks forward span	5 (5.15)	Not completed	3.46	9	-	55.55		
Verbal working memory	Backward digit span	4 (4)	4 (4)	3	8	-	50.00	50.00	0.00
Visuospatial learning ability	Corsi supraspan	14.27 (13.52)	Not completed	5.75	29.16	-	46.36		
Theory of mind	Story-based empathy task (SET)	18 (18.25)	18 (18.25)	16.12	18	-	100.00	100.00	0.00
Semantic memory	Pyramids and palm trees test (PPT)—picture version	52 (51.62)	52 (51.62)	40.15	52	-	100.00	100.00	0.00
	PPT—word version	51 (50.73)	52 (51.73)	40.78	52	-	98.08	100.00	+1.92
Attention	Attentional matrices	44 (43)	32 (31)	24	60	-	73.33	53.33	−20.00
Constructional apraxia	Clock drawing test	6.5 (6.5)	Not completed	6	10	-	65.00		
	Free hand-copying of drawings	12 (11.5)	12 (11.5)	8	12	-	100.00	100.00	0.00
General intelligence	Raven’s colored progressive matrices	33 (34.4)	Not completed	21.21	36	-	91.67		
Speed of processing	Trail making test (TMT)—A	33 (16)	39 (22)	94	94	-	24.09	28.47	+4.38
	TMT-B	110 (52)	83 (25)	283	283	-	46.61	35.17	−11.44
	TMT B-A	77(36)	44(3)	187	187	-	77.77	44.44	−33.33
General psychological well-being	Psychological well-being (PWB) scales	198	188	NA	252	-	78.57	74.60	−3.97
Functional communication and quality of life	Communication outcome after stroke (COAST-IT; useful in all conditions involving communication difficulties)	73	75	NA	80	-	91.25	93.75	+2.5

PM’s neuropsychological test scores upon admission (first assessment) and discharge from the hospital (second assessment). The 1^st^ column reports the cognitive domain targeted by each test. The 3rd column reports the scores at the First assessment. The 4th column reports the scores at the second assessment. In these two columns, scores corrected for age and education are in brackets and those falling below cut-off are marked with the symbol *. In contrast, the symbol ° marks those APACS tasks that reported a significant change at the second assessment (see 7th column). The 5th column reports the clinical cut-offs for each test. The 6th column reports the maximum score for each test, which was used to percentage-transform raw scores. The 7th column reports APACS thresholds for significant changes (the first value reports the threshold for a significant worsening, while the second the threshold for a significant improvement). 8th and 9th columns report the percentage scores, following the formula: (obtained score/maximum attainable score)*100. The 10th column reports the magnitude of the percentage score difference between the first and the second assessment. Negative values indicate declines in performance, while positive ones indicate improvements. Note that in some cases thresholds may not be available: this happens whenever the obtained score falls outside the range of normative sample scores (i.e., higher than maximum value or lower than minimum value) based on which the thresholds were estimated.

The patient showed no relevant impairment, except for a slight pragmatic deficit (see Table 1); for this reason, she took part in a rehabilitation program targeting pragmatic abilities, based on a modified version of the Pragmatics of Communication (PragmaCom) training (see Bambini et al., 2020b, 2022). About 2 weeks before discharge from the hospital, PM suffered a sudden worsening of her clinical conditions characterized by fatigue, marked generalized asthenia, nausea, psychomotor slowing, and optic neuritis (inflammation of the optic nerve involving pain, vision loss in one eye, visual field loss, and loss of color vision). These symptoms were treated with intravenous pulsed methylprednisolone. A magnetic resonance scan revealed diffuse cortical atrophy, enlarged ventricles, hyperintense alteration of the peri- and supraventricular white matter bilaterally, and demyelinating lesions in the spinal cord. However, the only previous scan dated back to 3 years before, making a comparison with the time before the worsening impossible. After the relapse, PM spent 2 days lying in bed because, besides physical symptoms and fatigue, she also felt very depressed and worried about the worsening of her clinical conditions, as she referred to the clinician. A second neuropsychological assessment was conducted before discharge, 3 days after the methylprednisolone administration (1 month after the first assessment, see Figure 1). At this point, PM reported persisting partial vision loss and eye pain, and concentration difficulties, which were qualitatively observed also by the experimenter. PM was not able to complete some tests (RAVLT—Delayed, Clock Drawing Test, Corsi span and supraspan, and Raven’s CPM) at the second assessment due to fatigue and was discharged shortly afterward.

Our aim was twofold: (i) to detect changes in performance in each test completed by PM at the second assessment, with respect to the performance in the same tests at the first assessment and (ii) to investigate which cognitive domains were mostly affected by the clinical worsening.

Usually, in neuropsychological case reports, changes in performance are simply detected by comparing raw test scores before and after a critical event (e.g., rehabilitation; Burdea et al., 2015; Boyd et al., 2016; Hara et al., 2017). This method, however, would not have fulfilled our second aim, because different tests have different score ranges. Therefore, to make tests comparable, we first percentage-transformed the obtained scores, so that they all referred to the same 0–100 range (Table 1, columns 8 and 9) according to the formula:


$$\frac{obtainedscore}{maximumattainablescore}\times 100$$

The same method has been previously used to compare performances (De Witte et al., 2019) or test scales with different score ranges (Klietz et al., 2019). After percentage transforming, we subtracted the second assessment percentage-transformed scores from the first assessment ones, thus obtaining the magnitude of each test’s change in scores between assessments (Table 1, column 10), which represents the change in performance after the clinical worsening. Since fluctuations in scores are expected between sessions, in the Results section, we will comment only on those percentage-transformed scores that exhibit a difference greater than 10% points between the two assessment times. In the Assessment of Pragmatic Abilities and Cognitive Substrates (APACS) test only, detection of changes could be examined more accurately by comparing the obtained scores with the available thresholds for significant changes provided in the test itself (column 7 of Table 1). If a value falls outside these thresholds, then a significant change (worsening or improvement) occurred. Note that for the Narratives task and APACS Total composite score, the lower threshold, indicating a significant worsening, is higher than the observed score at the first assessment; this can happen if a practice effect is expected. Scores in two APACS tasks (Interview and Figurative Language 2) were too low to be compared with the thresholds; therefore, for these tasks, we examined the magnitude of the percentage score difference.

To detect deficits in the performance in each test at both assessments, we relied on the inspection of clinical cut-offs (column 5 of Table 1). PM’s performance is illustrated in Table 1. Separate figures for pragmatics and other cognitive domains illustrate changes in performance and are built using the difference in percentage-transformed scores.

## Results

At the first assessment, PM showed a slight impairment in both APACS pragmatic comprehension and production (she scored below the cut-off in APACS Interview and Figurative Language 2 tasks, resulting in below cut-off composite scores for Pragmatic Production, Pragmatic Comprehension, and APACS Total). At the second assessment, comparisons with the thresholds for significant changes in APACS reported a significant worsening in almost all task scores (Description, Narratives, and Figurative Language 1) and composite scores (Pragmatic Production and APACS Total). All these scores, together with those of Interview, Figurative Language 2, and Pragmatic Comprehension, fell below the cut-off at second assessment, indicating a diffuse impairment. Even though thresholds for significant changes were not available for the low scores obtained by PM in Interview and Figurative Language 2, this latter task’s score exhibited a decline of 10% points, therefore worthy of consideration. Instead, PM scored significantly higher in humor comprehension, exhibiting an opposite tendency as compared to the other tasks. Qualitatively, during the second assessment, PM explicitly complained about difficulties in organizing her speech, resulting in short conversations with other people; she attributed these difficulties to increased fatigue and concentration problems, also reported by the clinician. PM also reported to the clinician that she felt very depressed and worried about the worsening of her clinical conditions. These feelings were probably reflected in the decrease in PWB score, which measured the patient’s general well-being.

**Figure 2:**
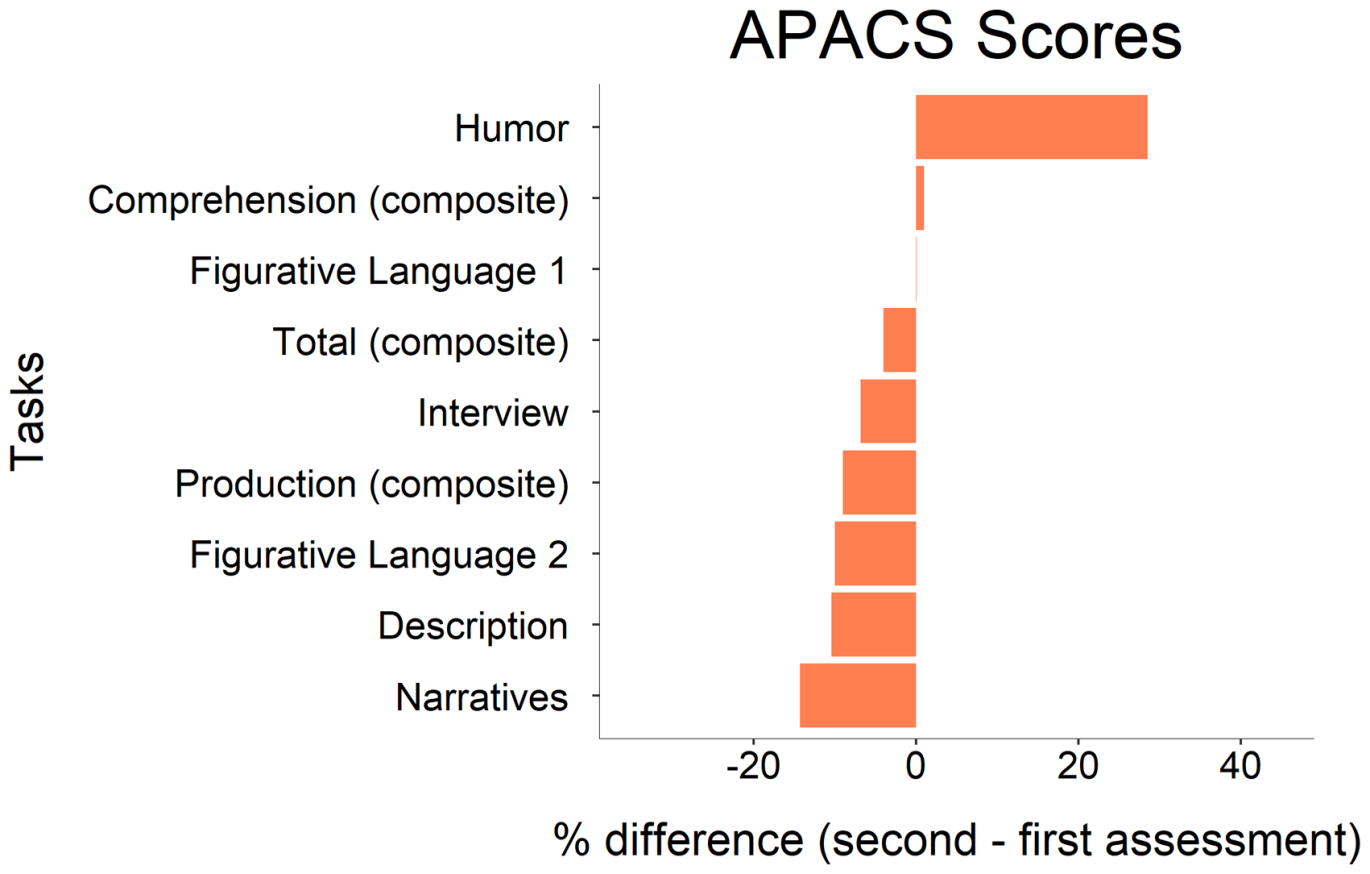
Difference between first and second assessment in APACS tasks performance. Raw scores have been converted into percentages following the formula: (obtained score/maximum attainable score)*100. Tasks are ordered according to the magnitude of changes, from the most positive to the most negative.

**Figure 3:**
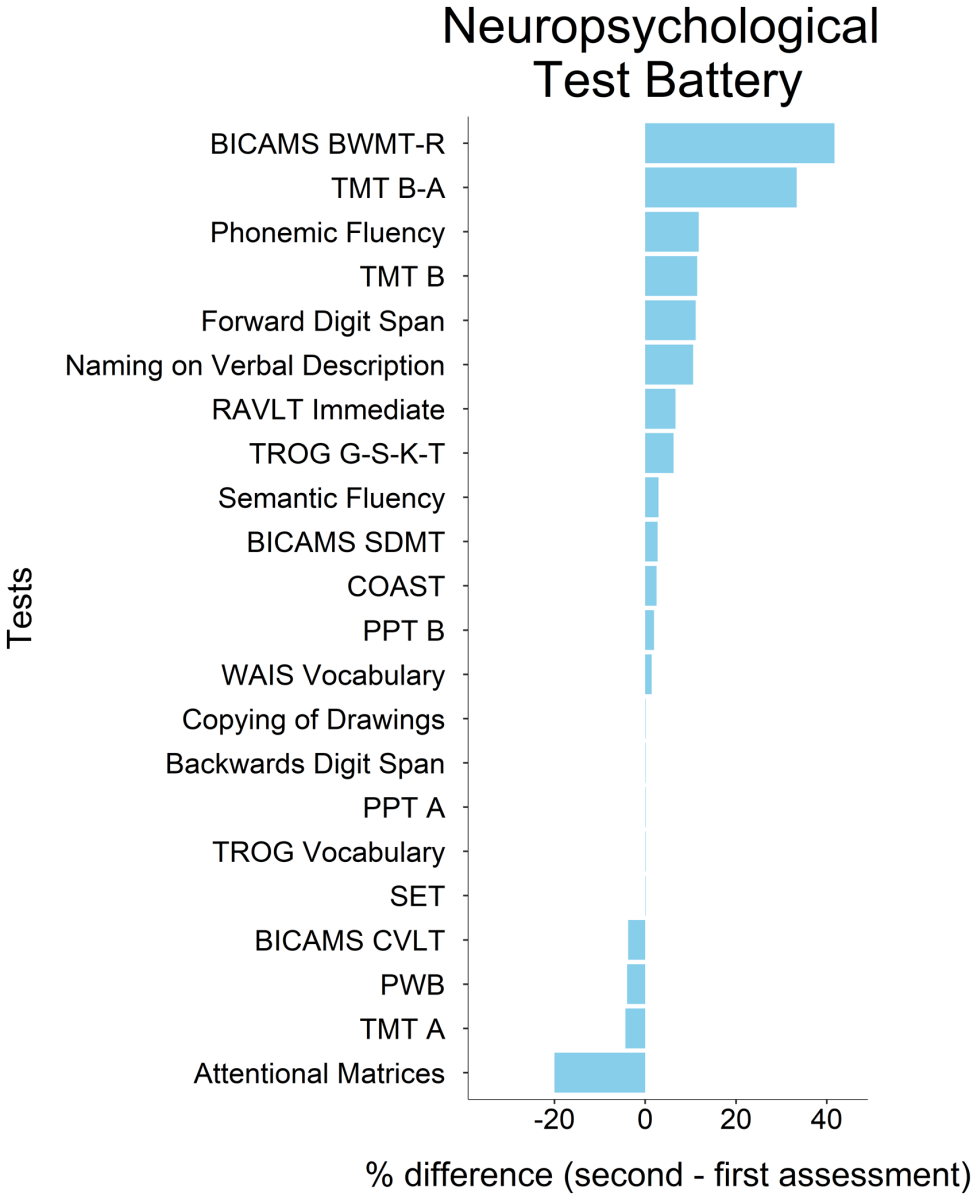
Differences in neuropsychological assessment percentage scores between first and second assessment. Raw scores have been converted into percentages following the formula: (obtained scores/maximum attainable score)*100. Tasks are ordered according to the magnitude of changes, from the most positive to the most negative. The direction of change in TMT scores has been reversed with respect to Table 1 to depict improvement and worsening straightforwardly, since a decrease in scores (negative magnitude) represents an improvement in this test. Tests’ acronyms are reported in Table 1.

No tests in the neuropsychological battery exhibited a comparable worsening, except for the Attentional Matrices (see Table 1; Figures 2, 3).

In some tasks, PM had a better performance at the second than at the first assessment, namely, on BICAMS BVMT-R, TMT B-A, Phonemic Fluency, TMT B, Forward Digit Span, and Naming on Verbal Description. On this latter task, PM’s performance was below the cut-off in both assessment sessions, indicating issues with lexical access.

In sum, PM exhibited a diffused, marked, and significant worsening in pragmatic abilities, accompanied by an analogous decline only in attention, while the rest of the cognitive profile remained stable.

## Discussion

This report illustrates the case of a patient, PM, who after a clinical relapse of MS symptoms showed a marked worsening, especially of pragmatic abilities, in the context of a stable cognitive functioning in almost all other neuropsychological tests.

Focusing on the performance in the pragmatic tasks from the APACS test, at the first assessment, PM scored below the cut-off in Interview and Figurative Language 2 and in Pragmatic Production, Comprehension, and Total composite scores, while at the second assessment, in addition to these tasks, also Description and Narratives were below the cut-off. Moreover, PM showed a significant worsening in almost all APACS tasks (Description, Narratives, Figurative Language 1 and 2, and composite scores Pragmatic Production and APACS Total), as indicated by the comparison with the thresholds for significant changes and by the magnitude of percentage score change, where the thresholds were not available. The qualitative analysis of the patient’s communicative abilities during clinical assessment highlighted difficulties in organizing speech that led PM to be under-informative, make abrupt topic shifts or lose verbal initiative, resulting in short conversations. PM also reported increased fatigue and concentration problems, noted by the clinician as well. The only pragmatic score that did not worsen and actually showed an improvement was Humor. This different behavior in the Humor task as compared to the other tasks could be related to the fact that Humor taps on different underlying cognitive substrates: factor analysis on the APACS scores in healthy controls composing the normative data suggested that performance on the Humor task is related to a different latent factor as compared the other APACS tasks (Arcara and Bambini, 2016). Alternatively, the improvement in Humor might reflect just some random fluctuations that are expected statistically when multiple tests are performed (Dunn and Kirsner, 2003), in line with the idea that Humor is primarily a pragmatic task (Bambini et al., 2020a).

At the cognitive level, the only neuropsychological test exhibiting a worsening comparable to the one in APACS was the Attentional Matrices, while several neuropsychological tests improved at the second assessment (BICAMS BVMT-R, TMT B-A, Phonemic Fluency, TMT B, Forward Digit Span, and Naming on Verbal Description), probably due to practice effects (note that for these tests, thresholds for significant change were not available).

The worsening in both pragmatics and attention is not surprising: significant relations between pragmatic and attentional/executive deficits in cognitively impaired MS patients have been previously reported (Carotenuto et al., 2018a). There is evidence of processing speed and attention impairment during relapses (Morrow et al., 2011) and mild executive dysfunction following acute use of corticosteroids (Prado and Crowe, 2019), as in the present case. Moreover, performance in Attentional Matrices is also influenced by psychomotor speed, which was found to be associated with communication abilities (Yap et al., 2022). More generally, several cognitive phenotypes regarding attention/executive functions, language, and multidomain deficits have been recently identified in MS patients and appear to be relevant for clinical purposes (De Meo et al., 2021). However, it does not seem that pragmatic impairment can be traced back to either attentional or psychomotor speed deficits. Indeed, in other tests involving attention and psychomotor speed (e.g., TMT-A, TMT-B), PM did not show a relevant worsening. As PM complained about issues with vision, it might also be that the declined performance in attentional matrices (with relatively small printed stimuli) was related to this peripheral issue, rather than to an attentional deficit *per se*.

Another aspect that deserves to be discussed is the potential role of mood in PM’s performance in cognitive tests. At the second assessment, a lower Psychological Well-Being (PWB) score was observed (see Table 1). This worsening in mood was also qualitatively noted by the clinician in PM’s behavior in the days before the second assessment (lying in bed for prolonged periods of time), strongly suggesting a depressive/apathic symptomatology. Although depression may have a role in PM’s worsening in attention and pragmatic abilities (Zurlo and Ruggiero, 2021), it is hardly to be the sole reason of the observed pattern of deficits. If depression had a major role, we would expect an effect extending not only to pragmatics but also to other aspects of cognitive functions that have been reported to be sensitive to mood in MS (as executive functions and verbal memory; Chiaravalloti and DeLuca, 2008; Mattioli et al., 2011), which were nevertheless spared in PM’s performance.

A similar argument can be used concerning a possible effect of fatigue, which also worsened in PM. Previous literature showed that fatigue and concentration difficulties are common symptoms in the MS population (Raimo et al., 2022). Although some authors reported an association between fatigue and cognitive performance (e.g., Takeda et al., 2021), the potential role of fatigue appears to be limited (Bol et al., 2010) or more relevant on sustained attention and alertness (Hanken et al., 2015). Again, the results did not support the interpretation according to which fatigue is a determinant factor in pragmatic worsening, as PM showed worsened performance in pragmatic tasks also at the beginning of the testing session and, conversely, good performance in some neuropsychological tasks, even if at the end of the testing session. Hence, no specific effect of sustained attention underlying the pragmatic impairment seems to emerge.

Taken together, there are two main interpretations of the overall results. On the one hand, it is plausible that PM developed a slight and widespread cognitive deficit where pragmatic abilities were particularly vulnerable. Within this interpretation, the pragmatic impairment would be associated with an attentional problem, although this emerged only in one task. Another interpretation is that PM developed a very selective impairment in some pragmatic abilities, and that the performance on the attentional task was influenced by irrelevant aspects with respect to cognitive functioning (i.e., the vision loss related to the clinical relapse). The MRI highlighted diffuse cortical atrophy and white matter lesions, which do not allow to hypothesize a precise correlation with cognitive deficits. Hence, with the available evidence, we cannot disentangle between these two interpretations, but we reported some arguments suggesting that, given the pattern observed across all administered tests, the pragmatic deficits of PM do not seem to be the mere consequence of other clinical symptoms (i.e., a deficit in attention, a change in mood, or fatigue).

Of relevance here is that, regardless of the underlying cause of PM’s observed cognitive performance, her pragmatic deficit was particularly susceptible to the clinical relapse and may have gone undetected by standard neuropsychological batteries focused on the traditional domains of language (e.g., vocabulary and grammar), attention, and memory. The present case thus highlights not only the importance of taking into account the possible impact of relapses and medication in the neuropsychological assessment of MS patients, but also the importance of investigating linguistic—including pragmatic—abilities in these patients, especially when they seem cognitively unimpaired but the clinician’s qualitative observations suggest a possible decline.

### Limitations

There are some limitations in the present study. First, there were few missing data from the second assessment, due to the patient’s clinical worsening. The patient was discharged soon after the second assessment, and it was not possible to perform further evaluations in order to examine the role of clinical worsening and corticosteroid administration and disentangle between different interpretations of the observed deficits. As a further methodological note of relevance for clinical purposes, most of the neuropsychological tests did not have available thresholds for detecting significant changes. This hampered the possibility of a solid statistical comparison of the effects across different tasks (which we partially overcame by comparing percentage score differences). The lack of thresholds for detecting significant changes is actually a widespread issue in Italian neuropsychological tests, and points to the necessity of investigating ways to reliably detect significant performance changes in the available tests (Aiello et al., 2021).

### Conclusion

We reported the case of a patient with MS who initially showed an isolated pragmatic impairment. After a worsening of the clinical condition, the patient’s performance showed a diffuse impairment in pragmatic tasks scores, but a relatively stable cognitive functioning as assessed by a standard neuropsychological battery. Our results suggest that pragmatic abilities are vulnerable and particularly susceptible to clinical worsening in MS, and that they might represent the pinnacle of a general cognitive impairment undetected by standard testing. In terms of implications for the clinical practice, the present case underlines the importance of considering pragmatic aspects during neuropsychological assessment in MS.

## Data availability statement

The raw data supporting the conclusions of this article will be made available by the authors, without undue reservation.

## Ethics statement

The study was reviewed and approved by Ethics Committee of Venice and San Camillo IRCCS Hospital. The patient provided her written informed consent to participate in this study. Written informed consent was obtained from the individual for the publication of any potentially identifiable images or data included in this article.

## Author contributions

SL: data collection, data analysis, and writing of the manuscript. GA: conception of the study, critical revision of the manuscript, and supervision of data analysis. VB and CS: critical revision of the manuscript and interpretation of the results. FB and MS: data collection and support to the clinical interpretation of the results. All authors contributed to the article and approved the submitted version.

## Funding

The work was supported by the Italian Ministry of Health under grant number GR-2018–12366092.

## Conflict of interest

The authors declare that the research was conducted in the absence of any commercial or financial relationships that could be construed as a potential conflict of interest.

## Publisher’s note

All claims expressed in this article are solely those of the authors and do not necessarily represent those of their affiliated organizations, or those of the publisher, the editors and the reviewers. Any product that may be evaluated in this article, or claim that may be made by its manufacturer, is not guaranteed or endorsed by the publisher.
