# Corrigendum: Case report: Pragmatic impairment in multiple sclerosis after worsening of clinical symptoms

**DOI:** 10.3389/fpsyg.2023.1137717

**Published:** 2023-01-30

**Authors:** Sara Lago, Francesca Bevilacqua, Maria Rosaria Stabile, Cristina Scarpazza, Valentina Bambini, Giorgio Arcara

**Affiliations:** ^1^IRCCS San Camillo Hospital, Venice, Italy; ^2^Department of Neuroscience, Padova Neuroscience Centre, University of Padova, Padua, Italy; ^3^Department of Neurology, ULSS2 Marca Trevigiana, Conegliano, Italy; ^4^Department of General Psychology, University of Padova, Padua, Italy; ^5^Department of Humanities and Life Sciences, University School for Advanced Studies IUSS, Pavia, Italy

**Keywords:** pragmatics, pragmatic impairment, multiple sclerosis, language, communication, case report

In the published article, there was an error regarding the affiliation(s) for author Cristina Scarpazza. As well as having affiliation 4, they should also have affiliation 1, IRCCS San Camillo Hospital, Venice, Italy.

The authors apologize for this error and state that this does not change the scientific conclusions of the article in any way. The original article has been updated.

