# Supplementary material for: Case report: Pragmatic impairment in multiple sclerosis after worsening of clinical symptoms
Source: Front Psychol. 2022 Nov 24;13:1028814. doi: 10.3389/fpsyg.2022.1028814 (PMC9731094; doi:10.3389/fpsyg.2022.1028814)
Supplement: Supplementary file 1 [file Data_Sheet_1.docx]

Supplementary Material

# Neuropsychological assessment tools

Neuropsychological assessment upon admission and before discharge from the hospital was conducted in four sessions lasting 1 hour each (8 in total). The assessment was conducted using the following instruments:

- Brief International Cognitive Assessment for Multiple Sclerosis (BICAMS; Italian version by Goretti et al., 2014) is one of the gold standards for the evaluation of cognitive deficits in MS (Amato et al., 2018). It includes the Symbol Digit Modalities Testing (SDMT, maximum score: 110), the California Verbal Learning Test-II (CVLT-II, maximum score: 80), and the Brief Visuospatial Memory Test-Revised (BVMT-R, maximum score: 36). These few specifically selected tests assess all the cognitive functions known to be impaired in MS: visual scanning, speed of processing, working memory, verbal and visuospatial learning, and memory.
- Assessment of Pragmatic Abilities and Cognitive Substrates (APACS; Arcara & Bambini, 2016) is a battery focusing on discourse production and non-literal language comprehension that consists of six tasks:
  - Interview assesses the ability to engage in a conversation through a semi-structured interview designed to elicit spontaneous speech. Discourse is rated according to a checklist evaluating the presence and frequency of several communicative difficulties in speech (e.g., repetition, incomplete utterances, echolalia), informativeness (over or under-informativeness, loss of verbal initiative), and information flow (missing referents, wrong order of the discourse elements, abrupt topic shift). Paralinguistic aspects are also considered (altered intonation, loss of eye contact, fixed facial expression, abuse of gestures) together with errors in grammar and vocabulary (anomias, paraphasias) since they have an impact on communication effectiveness. Each type of communication difficulty is rated 0/1/2 according to its frequency, corresponding to always/sometimes/never (maximum score: 44).
  - Description assesses the ability to share relevant information in everyday situations through the description of the main elements depicted in 10 photographs (presented one by one). For each element, a score is assigned, discriminating missed, partially correct, and correct identification (0/1/2; maximum score: 48).
  - Narratives measure the ability to comprehend texts of increasing length read aloud by the experimenter. Each story is followed by an open question about the global topic; some yes/no questions about specific elements of the story, either implicit or explicit, rated 0 or 1; two open questions requiring an explanation of non-literal expressions contained in the text and rated 0/1/2, based on the accuracy of the explanation (maximum score: 56).
  - Figurative Language 1 assesses the ability to infer non-literal meanings through multiple-choice questions. Fifteen non-literal expressions (five idioms, five metaphors, and five proverbs) are presented with a short context, together with three possible explanations, including the correct (figurative) interpretation, scored 1, and two incorrect interpretations scored 0 (maximum score: 15).
  - Humor assesses the ability to understand humorous statements. Seven brief stories are presented with three possible endings: a correct (funny) ending and two incorrect endings. The participant must select the ending functioning as the punchline of the story. Each item is scored 1 or 0 (maximum score: 7).
  - Figurative Language 2 assesses the ability to understand figurative expressions and articulate their meanings through verbal explanation (5 highly familiar idioms, 5 novel metaphors, and 5 common proverbs. Responses are rated 0/1/2 based on the accuracy of the explanation (maximum score: 30).
  - Three composite scores are derived from the APACS tasks: Pragmatic Production (from Interview and Description tasks), Pragmatic Comprehension (from Narratives, Figurative Language 1, Humor, and Figurative Language 2 tasks), and APACS Total (from Pragmatic Production and Comprehension scores).

Additional neuropsychological assessment covered the following areas:

- Language (Test of Reception of Grammar 2 (TROG-2) – blocks G, S, K, T, maximum score: 16; TROG-2 – Vocabulary, maximum score: 48, Bishop, 2003; WAIS Vocabulary, maximum score: 70, Orsini & Pezzuti, 2013);
- Executive functions supported by language (Naming on Verbal Description, maximum score 38; Phonemic and Semantic Fluency, maximum score: number of words that the subject produces for each category; Novelli et al., 1986);
- Verbal memory (Rey Auditory Verbal Learning Test, immediate and delayed; maximum score 75 and 15 respectively; Carlesimo et al., 1996);
- Short-term memory, verbal and visuospatial (Forward Digit Span and Corsi Blocks Forward Span, maximum scores 9 on each test; Monaco et al., 2013);
- Verbal working memory (Backwards Digit Span; maximum scores 8; Monaco et al., 2013);
- Visuospatial learning ability (Corsi supraspan; maximum score 29,16; Spinnler and Tognoni, 1987);
- Theory of Mind (Story-based Empathy Task (SET), maximum score: 18, Dodich et al., 2015);
- Semantic memory (Pyramids and Palm Trees Test – Picture (A) and Word (B) version, maximum score: 52, Gamboz et al., 2009).
- Attention (Attentional Matrices; maximum score 60; Della Sala et al., 1992);
- Constructional apraxia (Clock Drawing Test and free hand-copying of drawings; maximum score 10 and 12 respectively; Mondini et al., 2011; Carlesimo et al., 1996);
- General intelligence (Raven’s Coloured Progressive Matrices; maximum score 36; Carlesimo et al., 1996);
- Speed of processing (Trail Making Test - A and B; the score is represented by the number of seconds to complete the task; Giovagnoli et al., 1996);

Besides cognitive aspects, well-being and quality of life were also assessed, using:

- Psychological Well-Being scales (Italian version by Ruini et al., 2003). For this study, total scores (from 42 to 252) were considered, as a measure of global well-being;
- Communication Outcome after Stroke (Italian version, COAST-IT; Bambini et al., 2017) is a patient-centered measure of functional communication and its impact on quality of life. Originally developed for people with aphasia following stroke and traumatic brain injury, it can be useful also in other conditions involving communication difficulties; maximum score: 80.

**References**

Arcara, G., & Bambini, V. (2016). A test for the assessment of Pragmatic Abilities and Cognitive Substrates (APACS): Normative data and psychometric properties. *Frontiers in Psychology*, *7*(70), 1–13. <https://doi.org/10.3389/fpsyg.2016.00070>

Bambini, V., Arcara, G., Aiachini, B., Cattani, B., Dichiarante, M. L., Moro, A., Cappa, S. F., & Pistarini, C. (2017). Assessing functional communication: validation of the Italian versions of the Communication Outcome after Stroke (COAST) scales for speakers and caregivers. *Aphasiology*, *31*(3), 332–358. https://doi.org/dx.doi.org/10.1080/02687038.2016.1225275

Bishop, D.V.M. (2003). Test for Reception of Grammar: Version 2: TROG-2. Harcourt Assessment.

Carlesimo, G. A., Caltagirone, C., Gainotti, G., and the Group for the Standardization of the Mental Deterioration Battery (Fadda, L., Gallassi, R., Lorusso, S., Marfia, G., Marra, C., Nocentini, U., & Parnetti, L. (1996). The Mental Deterioration Battery: Normative Data, Diagnostic Reliability and Qualitative Analysis of Cognitive Impairment. *European Neurology*, *36*, 378–384. <https://doi.org/10.1159/000117297>

Della Sala, S., Laiacona, M., Spinnler, H., & Ubezio, C. (1992). A cancellation test: Its reliability in assessing attentional deficits in Alzheimer’s disease. *Psychological Medicine*, *22*(4), 885–901. https://doi.org/10.1017/S0033291700038460

Dodich, A., Cerami, C., Canessa, N., Crespi, C., Iannaccone, S., Marcone, A., Realmuto, S., Lettieri, G., Perani, D., & Cappa, S. F. (2015). A novel task assessing intention and emotion attribution: Italian standardization and normative data of the Story-based Empathy Task. *Neurological Sciences*, *36*(10), 1907–1912. <https://doi.org/10.1007/s10072-015-2281-3>

Gamboz, N., Coluccia, E., Iavarone, A., & Brandimonte, M. A. (2009). Normative data for the Pyramids and Palm Trees Test in the elderly Italian population. *Neurological Sciences*, *30*(6), 453–458. https://doi.org/10.1007/s10072-009-0130-y

Giovagnoli, A. R., Del Pesce, M., Mascheroni, S., Simoncelli, M., Laiacona, M., & Capitani, E. (1996). Trail Making Test: Normative values from287 normal adult controls. *Italian Journal of Neurological Sciences*, *17*(4), 305–309. https://doi.org/10.1007/BF01997792

Goretti, B., Niccolai, C., Hakiki, B., Sturchio, A., Falautano, M., Minacapelli, E., Martinelli, V., Incerti, C., Nocentini, U., Murgia, M., Fenu, G., Cocco, E., Marrosu, M. G., Garofalo, E., Ambra, F. I., Maddestra, M., Consalvo, M., Viterbo, R. G., Trojano, M., … Amato, M. P. (2014). The brief international cognitive assessment for multiple sclerosis (BICAMS): Normative values with gender, age and education corrections in the Italian population. *BMC Neurology*, *14*(1), 1–6. <https://doi.org/10.1186/s12883-014-0171-6>

Monaco, M., Costa, A., Caltagirone, C., & Carlesimo, G. A. (2013). Forward and backward span for verbal and visuo-spatial data: Standardization and normative data from an Italian adult population. *Neurological Sciences*, *34*(5), 749–754. <https://doi.org/10.1007/s10072-012-1130-x>

Mondini, S., Mapelli, D., Vestri, A., Arcara, G., & Bisiacchi, P. S. (2011). Esame neuropsicologico breve 2. Raffaello Cortina Editore.

Novelli, G., Papagno, C., Capitani, E., Laiacona, M., Vallar, G., & Cappa, S. F. (1986). Tre test clinici di ricerca e produzione lessicale. Taratura su soggetti normali. *Archivio Di Psicologia, Neurologia e Psichiatria*, *47*, 278–296.

Orsini A., Pezzuti L. (2013). WAIS-IV. Contributo alla taratura italiana. Giunti OS.

Ruini, C., Ottolini, F., Rafanelli, C., Ryff, C., & Fava, G. A. (2003). La validazione italiana delle psychological well-being scales (PWB). *Rivista Di Psichiatria*, *38*(3), 117–130. https://doi.org/doi 10.1708/173.1864
